# A sensitive flow cytometric methodology for studying the binding of *L. chagasi *to canine peritoneal macrophages

**DOI:** 10.1186/1471-2334-5-39

**Published:** 2005-05-24

**Authors:** Ricardo Gonçalves, Etel R Vieira, Maria N Melo, Kenneth J Gollob, David M Mosser, Wagner L Tafuri

**Affiliations:** 1 Faculdade de Medicina – DAPML – Universidade Federal de Minas Gerais (UFMG), Brazil; 2 Departamento de Bioquímica e Imunologia – Instituto de Ciências Biológicas (ICB), Universidade Federal de Minas Gerais (UFMG), Brazil; 3 Departamento de Parasitologia, Instituto de Ciências Biológicas (ICB), Universidade Federal de Minas Gerais (UFMG), Brazil; 4 Cell Biology and Molecular Genetics, University of Maryland, College Park, MD 20742, USA; 5 Departamento de Patologia Geral, Instituto de Ciências Biológicas (ICB), Universidade Federal de Minas Gerais (UFMG), Brazil

## Abstract

**Background:**

The *Leishmania *promastigote-macrophage interaction occurs through the association of multiple receptors on the biological membrane surfaces. The success of the parasite infection is dramatically dependent on this early interaction in the vertebrate host, which permits or not the development of the disease. In this study we propose a novel methodology using flow cytometry to study this interaction, and compare it with a previously described "in vitro" binding assay.

**Methods:**

To study parasite-macrophage interaction, peritoneal macrophages were obtained from 4 dogs and adjusted to 3 × 10^6 ^cells/mL. Leishmania (Leishmania) chagasi parasites (stationary-phase) were adjusted to 5 × 10^7 ^cells/mL. The interaction between CFSE-stained Leishmania chagasi and canine peritoneal macrophages was performed in polypropylene tubes to avoid macrophage adhesion. We carried out assays in the presence or absence of normal serum or in the presence of a final concentration of 5% of C5 deficient (serum from AKR/J mice) mouse serum. Then, the number of infected macrophages was counted in an optical microscope, as well as by flow citometry. Macrophages obtained were stained with anti-CR3 (CD11b/CD18) antibodies and analyzed by flow citometry.

**Results:**

Our results have shown that the interaction between *Leishmania *and macrophages can be measured by flow cytometry using the fluorescent dye CFSE to identify the *Leishmania*, and measuring simultaneously the expression of an important integrin involved in this interaction: the CD11b/CD18 (CR3 or Mac-1) β2 integrin.

**Conclusion:**

Flow cytometry offers rapid, reliable and sensitive measurements of single cell interactions with *Leishmania *in unstained or phenotypically defined cell populations following staining with one or more fluorochromes.

## Background

Leishmaniasis is a disease resulting from infection by protozoa of the genus *Leishmania*, which affects man and several other species of mammals. In the new world *Leishmania *is transmitted to man and animals by blood-sucking sandflies of the species *Lutzomyia longipalpis *[1-3]. Infection is initiated when the sandfly regurgitates infectious forms of promastigotes, termed "metacyclics" [4], into a small pool of blood formed by the blood-sucking female [5]. Upon inoculation of the host, promastigotes are phagocytized by skin macrophages, where they transform into aflagellar ovoid bodies known as amastigotes. In mammals, *Leishmania *are dimorphic obligate intracellular parasites, which reside and multiply mainly within the phagolysosomal compartment of mononuclear phagocytes [6-8]. Depending on the species, the parasite or infected macrophages, may spread and give rise to secondary infections in distant organs, producing distinct clinical forms of the disease [9]. Thus, the pathogenesis of *Leishmania *infections depends to a large extent on the relationship between the parasites and host macrophages.

The interaction of *Leishmania *promastigotes with mononuclear phagocytes has been characterized, and multiple receptor-ligand interactions have been implicated to play a role in the attachment to, and uptake of, promastigotes by macrophages [8]. These interactions include the binding of parasite surface molecules (lipophosphoglycan and gp63) or host derived opsonins (complement, fibronectin and immunoglobulin) to multiple macrophage receptors. The best characterized of these systems is the binding of serum complement opsonized promastigotes to macrophage receptors for complement [10]. Complement-dependent opsonization of parasites not only improves their adhesion to macrophages, but also enhances their intracellular survival [11]. Although the direct interaction of promastigotes with macrophages, in the absence of opsonization, has been well established for several species of *Leishmania*, the identity of the macrophage receptors involved in this interaction, and to a lesser extent, the ligands on the surface of promastigotes, remains unresolved [12]. The macrophage complement receptor Mac-1 (CD11b/CD18 or CR3) has been demonstrated to be crucial for the interaction of serum opsonized promastigotes with both human and murine macrophages [10,13-15].

In the literature, there are many "in vitro" methods describing the *Leishmania-*macrophage interaction. These techniques are performed quantifying parasites and macrophages over coverslips where parasites can be counted by conventional microscopy, immunofluorescent microscopy (immunolabelled parasites) or radioactivity associated with the cell lysates determined by a scintillation counter [11,14,16,17]. However, these techniques are laborious, time consuming, and do not allow an accurate characterization of the cells and surface receptors involved with parasite uptake. Moreover, most of these studies involve human or murine monocytes-macrophages and *Leishmania major*. "In vitro" studies using canine macrophages and *Leishmania chagasi *are rare in the literature. As far as we know, Marzochi et al. (1999) were the first to report binding-assays between different species of *Leishmania *and canine macrophages [18].

In this study we propose a flow cytometric method to study peritoneal canine macrophage-*Leishmania chagasi *interaction by a binding assay using the stable intra-cytoplasmic fluorocrome, 5,6-carboxyfluorescein diacetate succinimidyl ester (CFSE) [19]. This methodology is less laborious than cell-cultures techniques, and is not limited by the subjective and time-consuming nature of microscopy, necessary to calculate parasitism ratios. Moreover, when associated with conventional flow cytometry analysis, this technique also allows the phenotypic characterization of the infected cells and the identification of the cell surface molecules involved in parasite uptake. Lastly, this method allows for the analysis of large populations of cells rather than the limited number that must be analyzed using microscopy.

## Methods

### Animals

Four mongrel dogs of unknown age were obtained from the City of Sabará (Suburban area of Belo Horizonte, City) (City hall Zoonosis Department), MG, Brazil. All dogs were negative for *Leishmania *by direct immunofluorescence (RIFI) and complement fixation tests (RFC). The dogs received anti-helminth and anti-ectoparasite treatment. Animals were maintained in quarantine with food and water "ad libidum". All animal studies were performed under the guidance and approval of the institutes' animal welfare committee under the supervision of a certified veterinarian.

### Ethics approval

The experiment protocol using dogs, was approved by CETEA (COMITÊ DE ÉTICA EM EXPERIMENTAÇÃO ANIMAL – UFMG), by number 034/2004.

### Obtaining the Peritoneal cells

For obtaining the peritoneal macrophages the dogs were sacrificed using a lethal dose (0,3 ml/Kg) of T-61^® ^drug (Intervet). The animals were positioned in decumbency and the peritoneal cavity was disinfected using alcohol-iodine-alcohol and washed with sterile PBS (Phosphate-Buffered Saline). The peritoneal cavity was opened by a 10 cm incision along the medial line using a sterile scalpel. Next, 700 to 900 mL of sterile cold PBS was added to the cavity and mixed. The PBS was collected using a 60 mL sterile syringe and poured into several 50 mL conical centrifuge tubes on ice. Immediately the cells were centrifuged at 250 g, 4°C, 15 minutes and the supernatant was discarded. Washed peritoneal cell suspensions were adjusted to 3 × 10^6 ^cells/mL in culture medium (**D-10 **- DMM + 10% fetal calf serum (FCS) + L-glutamine + penicillin-streptomycin).

### Obtaining the parasites

*Leishmania (Leishmania) chagasi *parasites (stationary-phase) were adjusted to 5 × 10^7 ^cells/mL in "phagocytosis buffer" culture medium (equal parts of Dulbecco's Modified Eagle Medium and Medium 199 supplemented with1% of BSA and 12,5 mM HEPES) [14].

The CFSE (carboxyfluorescein diacetate succinimidyl ester – Molecular Probes C-1157) dye was used as previously described [19]. Briefly, parasites were washed twice, in PBS 1,200 g, 10 minutes, 4°C. Parasites were resuspended in 1 mL of PBS (5 × 10^7 ^parasites/mL) with × nM of CFSE (using a stock of 2.8 μg/mL in DMSO) and incubated in a 37°C water bath for 10 minutes in the dark.. After this, they were washed in 10 mL of cold PBS + 10% of fetal bovine serum and centrifuged at 1,200 g. The pellet was diluted in phagocytosis buffer culture medium protected in the dark.

We have tested the viability and vitality of the CFSE-stained *Leishmania*, by observing their mobility under the light microscope. The fluorescence was confirmed by observing stained *Leishmania *using a fluorescent microscope. Dyed parasites were also used to infect mice (BALB/c) and hamsters to observe their virulence. The spleen and liver were collected after 4 weeks of infection and slides were prepared for analysis using light microscopy. The results indicated that CFSE stained parasites have an equivalent infective capacity as non-stained parasites (data not shown).

### Leishmania binding assay

The interaction between CFSE-stained *Leishmania-chagasi *and canine peritoneal macrophages was performed in polypropylene tubes to avoid macrophage adhesion. 100 μL of macrophages (3 × 10^5 ^cells) and 100 μL of CFSE stained-*Leishmania chagasi *(5 × 10^6 ^cells) were combined in the tubes and maintained for 45–60 minutes at 37°C in a 5% CO_2 _environment.

We carried out assays in the presence of normal serum or in the presence of a final concentration of 5% of C5 deficient (serum from AKR/J mice) mouse serum [20].

### Specific staining with anti-CR3 (CD11b/CD18)

Macrophages obtained as described above were stained with anti-CR3 (CD11b/CD18) rat anti-Human CD18-RPE antibody (Serotec) that shows cross reactivity with canine cells. Non-conjugated, purified Mouse anti-Canine CD11b (Serotec) conjugated using the Kit Zenon tricolor (Molecular probes – Z-25080) as described by the manufacturer with the "C" compound that represent a 647 nm emission band and can be used with CFSE and R-PE. Cells were incubated with labeled antibody solutions for 20 min at 4°C. After staining, preparations were washed with 0.1% sodium azide PBS, fixed with 200 μl of 2% formaldehyde in PBS and kept at 4°C until data were acquired using a flow cytometer (FACSVantage, Becton & Dickinson, San José, CA, USA).

### Flow cytometry analysis

The cells were run on an analytical flow cytometer equipped with a laser emitting at 488 nm (FACSVantage, Becton-Dickinson, San Diego, CA, USA). Whole cells were excluded from fragments by gating based on the forward and side scatter signals. CFSE-stained promastigotes bound to macrophages were detected according to their relative fluorescence intensities using the FL1 (green) detector as compared to those of uninfected cells. Both the frequency of cells associated with Leishmania, as well as the intensity of cells associated with Leishmania was determined. Analyses were performed on 40,000 to 100,000 gated events, and numeric data were processed with WinMDI software version 2.8 (Joseph Trotter: ).

In order to confirm the intensity of macrophages binding with dyed *Leishmania*, we did an experimental "in vitro" assay using different macrophage/ parasite in ratios of : 1:1, 1:2, 1:5, 1:10, 1:15, 1:20 and 1:30 Macrophages:*Leishmania*).

### Statistical analysis

Results are given as complete randomized design and the means from each group were compared using "student t test". P value less than 0.05 was considered significant.

## Results

**Figure 1:**
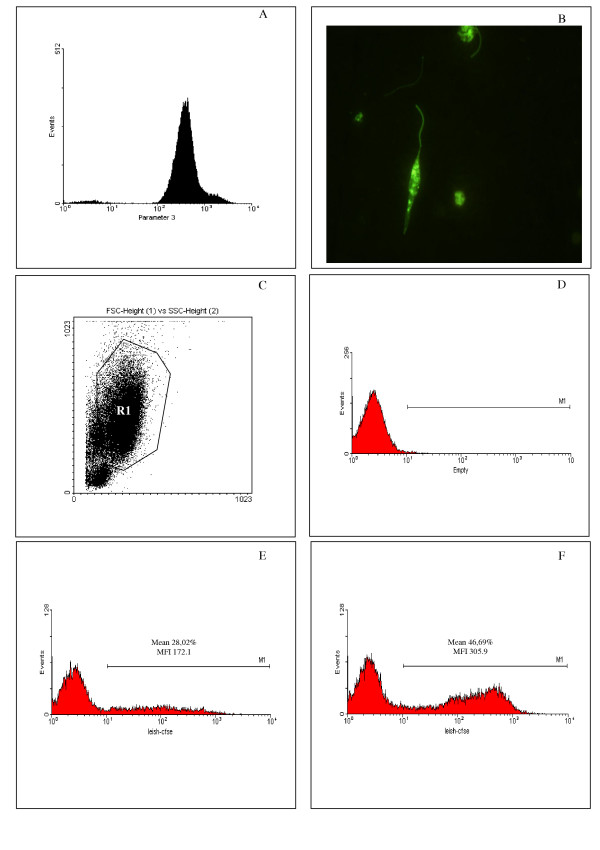
The use of CFSE stained- *L. chagasi *to evaluate parasite-canine macrophage interaction. *L. chagasi *promastigotes were stained with the intracellular dye CFSE and the efficiency of the staining procedure evaluated trough flow cytometry (A) and conventional fluorescence microscopy (B). CFSE-stained parasites were used for *in vitro *infection of peritoneal dog macrophages (X to Y ratio) and the parasite-macrophage interaction was evaluated through flow cytometry. Macrophages were selected based on forward- versus side-scatter parameters (C) and the frequency and intensity of CFSE+ macrophages was evaluated (D to F – MI Marker). At least 30,000 events were collected. (D) Non-infected macrophages. (E) Macrophages infected by CFSE-stained parasites in absence of C5 deficient sera. (F) Macrophages infected by CFSE-stained parasites in presence of C5 deficient sera. Data represents the mean ± SE of the frequency of infected macrophages and the CFSE fluorescence intensity of infected macrophages, obtained for one dog, representing identical experiments of four dogs.

Flow cytometry was used to analyze the interaction between canine peritoneal macrophages and *L. chagasi *promastigotes, stained with the fluorescent dye CFSE (Fig. 1), and compared to a conventional methodology ("in vitro" binding-assays). Experiments were carried out to evaluate the frequency and intensity of *Leishmania *bound macrophages using gates designed to encompass the macrophage population and further identifying CFSE positive (*Leishmania *bound macrophages) and CFSE negative (*Leishmania *unbound macrophages) populations. Aliquots of CFSE-stained parasites were evaluated using the flow cytometer to analyze the efficiency of CFSE staining. More than 95% of the parasites were strongly and homogeneously stained by CFSE, and non-stained parasites were easily distinguished (Fig. 1A, 1B). To evaluate the macrophage-parasite interaction, macrophages were selected based on the forward and side scatter profile, depicted in Figure 1C (region R1), excluding lymphocytes (lower left hand population). Macrophages bound, or not, with *Leishmania *were easily distinguished following 1 hour of interaction using flow cytometric analysis and detection of CFSE fluorescence with the FL1 (green) detector (Fig. 1E and 1D, respectively). The Leishmania bound macrophages are identified by the M1 marker (Fig. 1E,1F), and position themselves to the right of the negative macrophages which are defined by control cultures of macrophages (Fig. 1D) in the absence of CFSE stained *Leishmania*. More than one population of macrophage-*Leishmania *conjugates are seen within the M1 marker and represent different intensities of association. The frequency and intensity of macrophages bound with *Leishmania *were increased when a C5 deficient mouse sera was used during the macrophage-*Leishmania *interaction (Fig. 1F). Free parasites present in the cultures were not detected during our analysis of infected macrophages, due to the fact that they are gated out of the analysis region based on size and granularity which puts them in the extreme lower left region of a FSC vs. SSC plot (data not shown).

**Figure 2:**
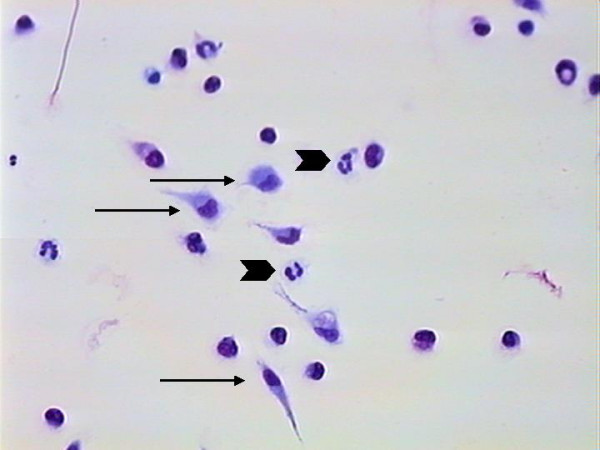
Cells from peritoneal lavage adhered to coverslips. The image shows the predominance of macrophages (arrow) and neutrophils (arrow head). Giemsa stain – 400×.

**Figure 3:**
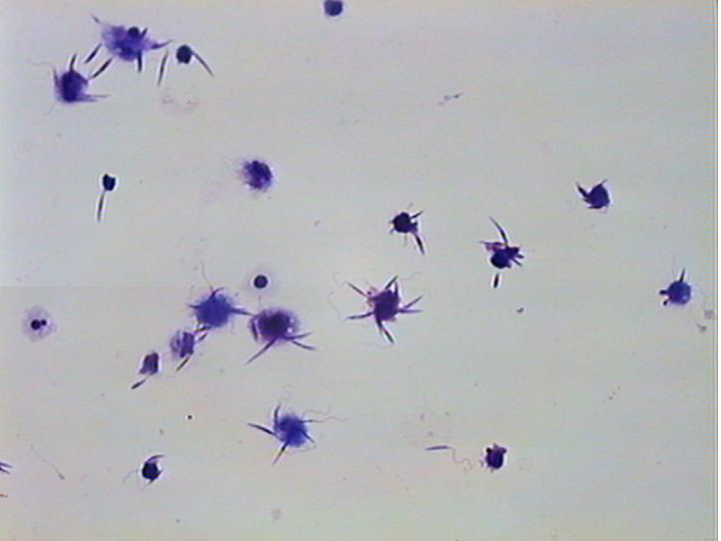
Conventional Binding assay showing the interaction of macrophages from one dog with several *Leishmaniae*. Giemsa stain – 400×.

**Figure 4:**
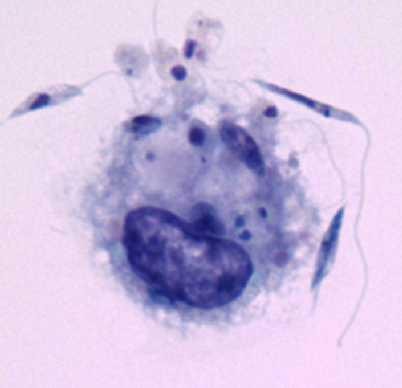
Conventional Binding assay showing the interaction of one macrophage with several *Leishmaniae*. Giemsa stain – 1000× optical microscope – 2× digitalized image.

**Figure 7:**
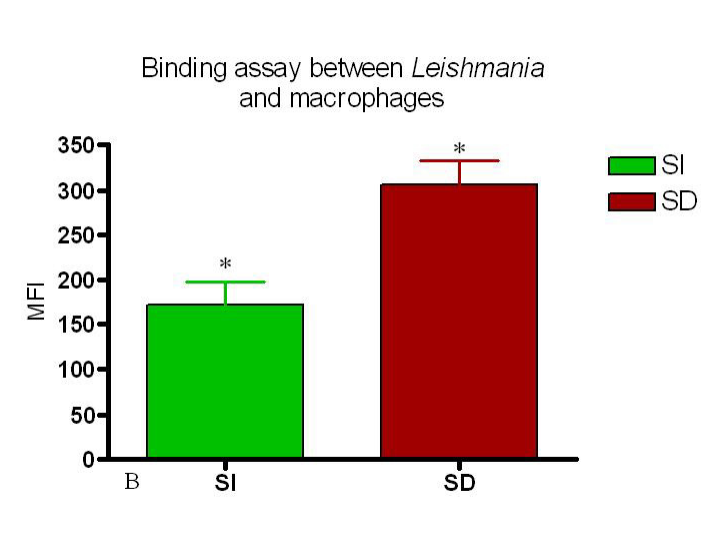
Serum dependent (SD) conditions induce a higher association of *Leishmania *with macrophages as measured by both mean fluorescent intensity and percent macrophages associated with *Leishmania*. This figure shows the MFI of serum independent SI and SD. analysis. n = 4 animals. * = p < 0.05.

**Figure 8:**
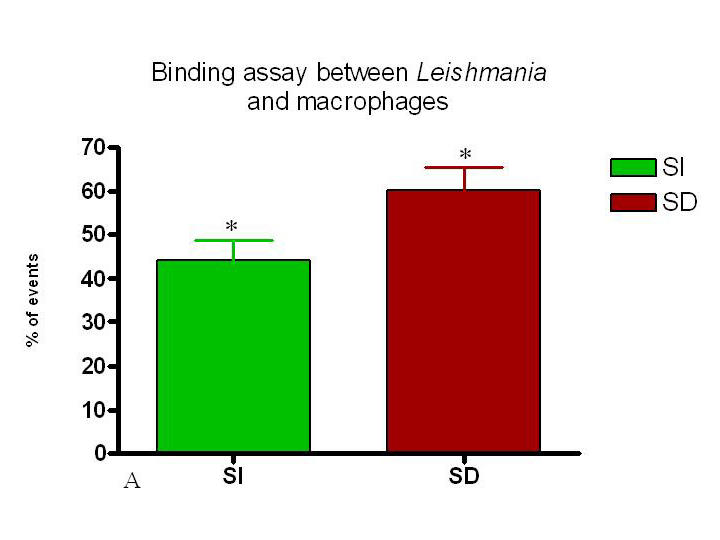
Percent of CFSE positive macrophages from SI and SD cultures. Canine macrophages were incubated for 1 hour with CFSE labeled *Leishmania *and analyzed using flow cytometry as described in Materials and Methods. n = 4 animals. * = p < 0.05.

Simultaneously, *Leishmania-*binding assays were carried out using 24-well plastic plates covered with coverslips, previously described by others [13,14]. Through microscopic morphologic analysis of the cells, the populations harvested from the canine peritoneum was found to be composed of mostly macrophages, monocytes and neutrophils (Fig. 2). Using a *Leishmania-*binding assay (Fig. 3 and 4) it was determined that macrophages show parasite binding after 1 hour of macrophage-*Leishmania *interaction and that the use of C5 deficient sera (serum dependent system) markedly increased the parasite burden. Serum dependent (SD) "in vitro" binding-assays showed an average of 2.43:1 (*Leishmania*:macrophage) in contrast to the serum independent (SI) assays which showed a lower average of 1.7:1. In addition, the numbers of macrophages bounded to *Leishmania *were higher in the SD assays than in the SI assays as seen by 37.41% and 11.41% macrophages bound to *Leishmania*, respectively. Finally, the increased interaction of *Leishmania *with macrophages when using SD as compared to SI conditions was also seen by flow cytometric analysis of the mean fluorescent intensity (MFI) and % bound macrophages (Fig. 7 and 8). Both MFI and percent macrophages bound with *Leishmania *were higher under the SD conditions (Fig. 7 and 8).

**Figure 5:**
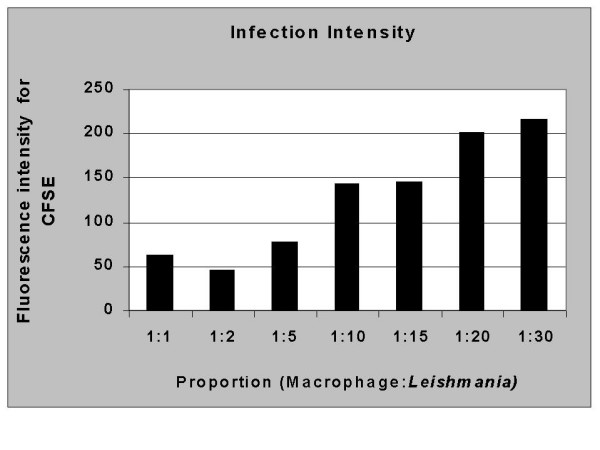
Increased parasite/macrophage ratios leads to an increased intensity of macrophages associated with CFSE marked parasites. The intensity of fluorescence increases in accordace with the number of parasites per macrophage used. Macrophages and parasites were combined as described in Materials and Methods and analyzed for fluorescent intensity using flow cytometry. The data represent the mean flourescent intensity of posive macrophage populations from cultures at each ratio studied. These studies are representative of 4 experiments.

Next to determine if increased numbers of CFSE-stained parasites would lead to a corresponding increase in the intensity of macrophage/parasite-CFSE pairs, a number of ratios of macrophage to parasite combinations were studied. As seen in Figure 5, an increased parasite/macrophage ratio leads to an increase in the mean fluorescent intensity (MFI) of the positive macrophage population.

### Evaluation of CR3 expression using flow cytometry

**Figure 6:**
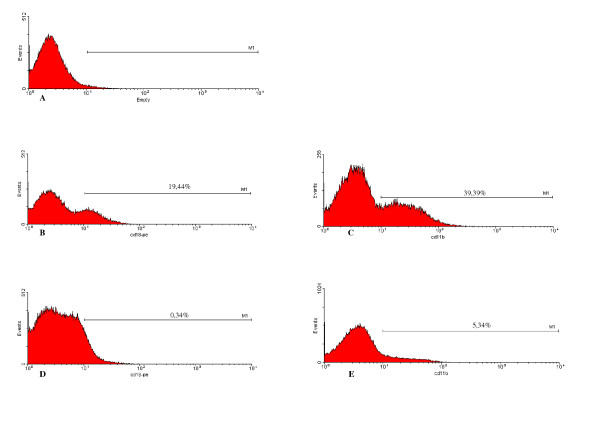
The levels of CD11b and CD18 fall dramatically following incubation with Leishmania under serum dependent (SD) culture conditions. Peritoneal macrophages were selected based on forward- versus side-scatter parameters as described in Materials and Methods and Figure 1 using flow cytometry. A) Isotype control with macrophages showing delineation of the M1 marker based on the negative population. B and C represent expression of CD18 (B) or CD11b (C) before incubation with *Leishmania*. D and E represent expression of CD18 (D) or CD11b (E) after association with *Leishmania *in a SD assay. In all cases the percent positive cells within the M1 marker are identified. This Figure is a representative diagram of 4 experiments with similar findings.

The expression of CR3 (CD11b/CD18) by peritoneal macrophages was determined using flow cytometry before and after interaction with *Leishmania *under both the SD and SI conditions (Figure 6).

**Figure 9:**
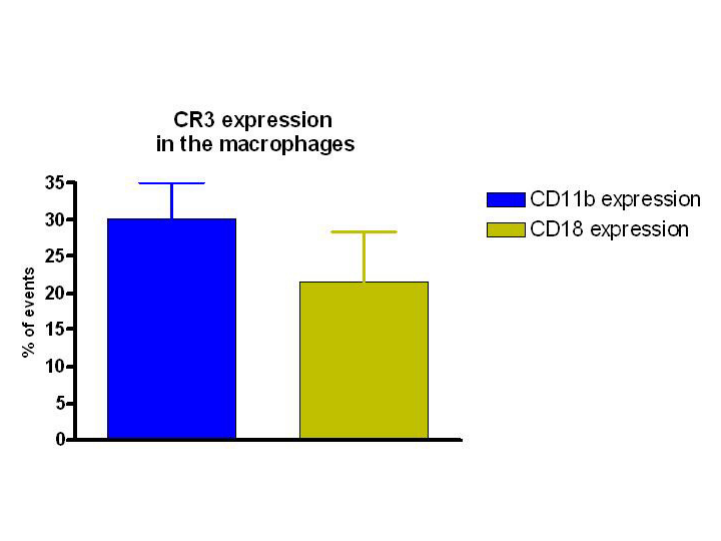
Percent peritoneal cells expressing CR3 as determined by expression of CD11b and CD18. Peritoneal cells from 4 dogs were stained ex vivo and analyzed using flow cytometry as described in Materials and Methods.

**Figure 10:**
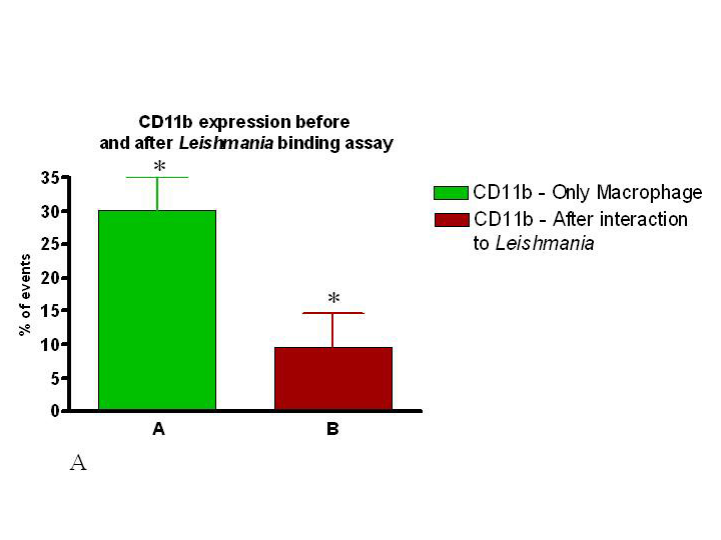
The expression of both CD11b and CD18 is dramatically reduced following association of peritoneal macrophages with *Leishmania *in both assays (SI or SD). Percent of CD11b expressing macrophages before and after incubation with *Leishmania *(SD assay).

**Figure 11:**
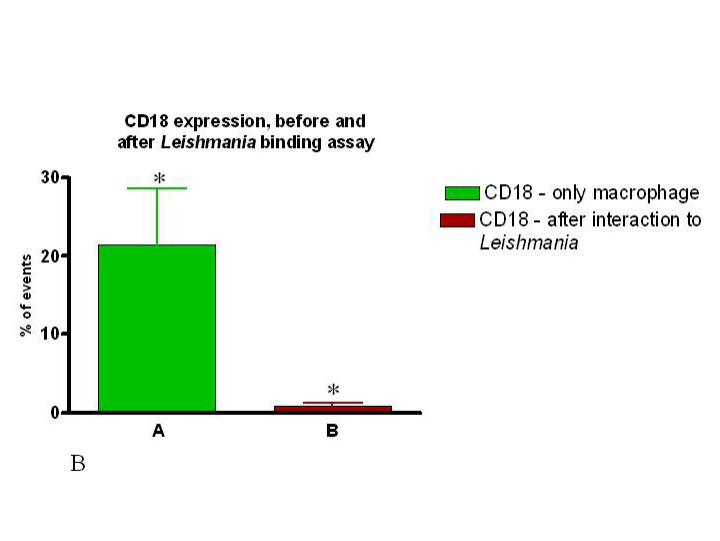
The expression of both CD11b and CD18 is dramatically reduced following association of peritoneal macrophages with *Leishmania *in both assays (SI or SD). Percent of CD18 expressing macrophages before and after incubation with *Leishmania *(SD assay). Macrophages were incubated with or without *Leishmania *for one hour and stained with the appropriate antibody followed by analysis using flow cytometry as described in Materials and Methods. n = 4 animals, and * p < 0.05.

There was no significant difference in the percent cells expressing CD11b versus those expressing CD18 amongst all the samples analyzed (Fig. 9). Interestingly, upon interaction with *Leishmania*, the frequency of macrophages expressing CD11b or CD18 dropped significantly in both the SI and SD conditions (Fig. 10 and 11). However, no difference in the expression of CD11b/CD18 was seen between the SI and SD conditions (data not shown).

## Discussion

American Visceral Leishmaniasis (AVL) is a zoonosis with dogs representing the principal domestic reservoir of the disease. AVL is present in at least a dozen Latin American countries, with 90% of human cases reported from Brazil, especially the north-eastern region [1-3,18,21,22]. Canine Visceral Leishmaniasis (CVL) appears to be spreading further in Brazil, and outbreaks have occurred in major Brazilian cities in the northeast of the country, such as Teresina, (PI), São Luiz (Maranhão), Fortaleza (Ceará), Salvador (Bahia), [23,24] and also in the southeast as in Belo Horizonte, [25,26] and Rio de Janeiro (RJ) [18]. These urban or predominantly peri-urban outbreaks appear to be increasing, but historically they are cyclical and recur following several year intervals of low endemicity. Domestic dogs have been the target of intense research concerning leishmaniasis with hopes of improving control of infected animals, and due to findings that show common clinical, immunological and pathologic aspects with human visceral disease. [27-34].

Macrophages are the main cells involved in the pathogenesis of the disease and they represent an ideal target cell population for "in vitro"studies. However, data concerning the behaviour of canine macrophages from naturally or experimentally infected animals with *Leishmania chagasi*, "in vitro", are limited. In this work we have demonstrated a reliable and practical method to quantify the frequency and intensity of host cells bound with *Leishmania*. This methodology is less laborious and more quantitative than cell-cultures techniques, removing the disadvantages of subjectivity and the time-consuming nature of microscopy when establishing parasitism ratios.

Our results, using the conventional *Leishmania*-binding assay, have confirmed previous work in the literature [35] where it has been demonstrated that the number of parasites bound to macrophages was dramatically increased when using serum containing complement. Importantly, these same results were obtained using analysis by flow cytometry with CFSE stained *Leishmania*. Moreover, a strong correlation was seen between both conventional microscopy based methods and our flow cytometry based approach.

In addition, this methodology permits the simultaneous use of conventional flow cytometry analysis to quantify the expression of membrane receptors related to *Leishmania*-macrophage interactions. For example, we have performed assays to determine the expression of CD11b/CD18 (CR3) [14,36] integrins involved in the interaction of *Leishmania *promastigotes with canine macrophages. Moreover, the MFI of macrophages associated with *Leishmania *increased with higher parasite/macrophage ratios, indicating a direct correlation between the number of parasites and the intensity of interaction with the host macrophage. Finally, other phenotypic markers can be used to clearly characterize the infected population and to describe functional characteristics of cell subpopulations through the analysis of cytokines and activation markers.

Our results have shown that CR3 (CD11b/CD18) receptor expression is lower in the presence of promastigote forms of *Leishmania*. Since we have performed binding-assays (not infection assays) we can consider that these receptors are either occupied by *Leishmania *binding, or that the receptor complexes have been internalized. Future "in vitro" studies are being carried out in our laboratory to resolve this question.

Several studies have been designed to study the interaction of parasites and macrophages. The majority used dead microorganisms (bacteria or fungi), inert or fluorescent particles (latex/polystyrene beads or zimozan), lamb's hematia, or finally, microorganisms labelled with antibodies [37]. The use of antibody labelled-living *L. amazonensis *was shown by Bertho et al (1992) as a poor method, because labelling antibodies were shed too quickly [38]. We have found a staining method using living-parasites that can be used without interfering with the interaction between macrophages and parasites, and it represents a stable association of the fluorescence with the parasite. The stability of the CFSE staining has been largely employed in other studies using living cells [39-42], and was recently used for staining and tracking the association of *T. cruzi *with host cells as well [43].

While CFSE is typically used in proliferation assays [44], our novel studies have defined another use for this marker to analyze the interaction between a parasite and host cells.

## Conclusion

In this work we propose a method for the study of the interaction between live *Leishmania *and macrophages using flow cytometry. Moreover, we have also demonstrated a system that can be useful for studies of the interaction of *L. chagasi *with canine peritoneal macrophages. These techniques open the door to many future studies designed to further investigate the interaction of *Leishmania *with host cell populations. Finally, CFSE stained *Leishmania *have been shown to be stable for many days, and could be used to study phagocytosis or *Leishmania-*survival.

## Competing interests

The author(s) declare that they have no competing interests

## Authors' contributions

RG conceived and designed the study, carried out the assay development, data analysis, and wrote the first draft of manuscript. ER participated in cytometry assay, cytometry analysis and critical review of the manuscript. MNM provided the parasite *Leishmania (Leishmania) chagasi*. KJG provided expert input for cytometry assay, critical review of manuscript and supervised the cytometry study. DMM provided expert critical review of manuscript. WLT carried out the *Leishmania *binding assay, provided expert input for writing and supervised the study. All authors have read and approved the final manuscript.

## Pre-publication history

The pre-publication history for this paper can be accessed here:


